# Protective Effects of Lignin-Carbohydrate Complexes from Wheat Stalk against Bisphenol a Neurotoxicity in Zebrafish via Oxidative Stress

**DOI:** 10.3390/antiox10101640

**Published:** 2021-10-18

**Authors:** Jie Gu, Min Guo, Liping Zheng, Xiaogang Yin, Linjun Zhou, Deling Fan, Lili Shi, Caoxing Huang, Guixiang Ji

**Affiliations:** 1 Nanjing Institute of Environmental Sciences, Ministry of Ecology and Environment, Jiangwangmiao Street 8, Nanjing 210042, China; gujie@nies.org (J.G.); guomin@nies.org (M.G.); zlp@nies.org (L.Z.); zhoulj@nies.org (L.Z.); fdl@nies.org (D.F.); sll@nies.org (L.S.); 2 Co-Innovation Center for Efficient Processing and Utilization of Forest Resources, College of Chemical Engineering, Nanjing Forestry University, Longpan Road 159, Nanjing 210037, China; yinxiaogang@njfu.edu.cn

**Keywords:** bisphenol A (BPA), wheat stalk lignin-carbohydrate complexes, zebrafish, antioxidant extract, oxidative stress

## Abstract

Lignin-carbohydrate complexes (LCCs) from different lignocellulosic biomass have shown biological qualities as antioxidant and immunostimulant. By contrast, the application of LCCs as protectant against neurotoxicity caused by different compounds is scarce. In this work, two kinds of LCCs with carbohydrate-rich and lignin-rich fractions were obtained from wheat stalk and used to protect against BPA-neurotoxicity in zebrafish. The results showed that BPA at a concentration of 500 µg/L results in neurotoxicity, including significant behavioral inhibition, and prevents the expression of central nervous system proteins in transgenic zebrafish models (Tg (HuC-GFP)). When the zebrafish was treated by LCCs, the reactive oxygen species of zebrafish decreased significantly with the change of antioxidant enzymes and lipid peroxidation, which was due to the LCCs’ ability to suppress the mRNA expression level of key genes related to nerves. This is essential in view of the neurotoxicity of BPA through oxidative stress. In addition, BPA exposure had negative effects on the exercise behavior, the catalase (CAT) and superoxide dismutase (SOD) activity, and the larval development and gene expression of zebrafish larvae, and LCC preparations could recover these negative effects by reducing oxidative stress. In zebrafish treated with BPA, carbohydrate-rich LCCs showed stronger antioxidant activity than lignin-rich LCCs, showing their potential as a neuroprotective agents.

## 1. Introduction

Plastic products, such as water bottles, milk bottles, and cans, are essential as food containers in our daily life. Bisphenol A (BPA) is one of the most used raw material for the manufacture of plastic containers [1]. However, BPA has been identified as an endocrine disruptor chemical, which can be toxic to the nervous, reproductive, and cardiovascular system [2,3]. Terrifyingly, BPA has even been detected in follicular fluid, maternal amniotic fluid and serum, placental tissue, and newborn urine [4,5]. Hence, there are growing concerns regarding its biosafety and toxicity in humans.

Many in vitro and in vivo studies revealed that BPA can disrupt the functions of various cells in the nervous system. For instance, Miyatake et al. (2006) [6] reported that the inherent cellular structure and biofunction of neuronal cells was significantly altered following BPA treatment. Recently, BPA was found to affect hypothalamus growth, leading to hyperactive behavior, in a zebrafish model [7]. Presumably, the key pathway for BPA-induced inhibition of cell function during neurogenesis is its ability to disrupt the protein and lipid structures via free radical-mediated mechanisms [2]. Hence, various antioxidants have been tested in animal models to minimize the neurotoxic effects of BPA at the cellular level. Antioxidants in plant extracts have been used as nutraceuticals to prevent or treat free radical-related diseases [8]. To minimize the neurotoxic effects of BPA, a natural antioxidants, such as α-lipoic acid (ALA), has been used as neuroprotective agents for reducing oxidative damage in a hippocampal cell line, an Alzheimer’s disease model, and an astrocyte cell line in vivo [9]. However, these natural antioxidants are derived from specific plants, most of which are rare and difficult to obtain in the wild.

Lignocellulosic biomass is an inexhaustible feedstock material containing cellulose, hemicellulose, lignin, and extractives [10,11]. Depending on various structural and functional groups, the antioxidant activity of lignin has been demonstrated, which can be applied in various biofields [12,13]. In plant cell wall, lignin and carbohydrates (mainly hemicellulose) are covalently linked to form the lignin–carbohydrate complexes (LCCs). Owing to the presence of both lignin and carbohydrates, LCCs possess antioxidant activity derived from lignin as well as high compatibility and adhesiveness to the cell because of the biological interaction between polysaccharides and cellular surface [14,15]. In this context, Dong et al. (2020) [15] reported that the LCCs extracted from various biomass materials exhibited excellent antioxidant activity against reactive oxygen species (ROS) both in vitro and in vivo. However, no study has evaluated whether LCCs can serve as neuroprotective agents to minimize the detrimental effects of ROS generation.

Cell lines are the common models to evaluate the efficacy of antioxidants in reducing BPA neurotoxicity. In addition, animal models such as zebrafish and mice have been proposed to further understand the efficacy of antioxidants against toxic chemicals [16]. In these animal models, the protective effects of antioxidants against BPA-induced neurotoxicity in the hypothalamus and cerebrum can be visually examined. Currently, zebrafish have emerged as a powerful in vivo animal model in pharmacological and toxicological research [17]. Interest in zebrafish models can be attributed to their specific properties such as small size and transparent body at the early developmental stages, which enable the clear observation of structural and functional changes in specific tissues following exposure to a certain chemical [18,19]. Although zebrafish models have been used to evaluate BPA neurotoxicity, this is the first time that zebrafish has been used as an in vivo model to assess the protective effect of LCCs against BPA neurotoxicity.

To this end, in this study, LCCs from wheat stalk (WS) (LCC-WS-A) were isolated and subjected to a short hydrothermal treatment to obtain complexes with a lower carbohydrate proportion (LCC-WS-B) and the association between LCC structure and its antioxidant properties was evaluated. The composition and structure of these two LCC preparations were comprehensively analyzed using high-performance anion-exchange chromatography (HPAEC) and nuclear magnetic resonance (NMR), respectively. Moreover, the antioxidant properties of these LCC preparations were evaluated based on free radical scavenging assays in vitro and antioxidant activity against BPA-induced ROS in a zebrafish model in vivo. The protective effects of LCCs against BPA-induced neurotoxicity were assessed by analyzing the hatching rate, behavior, and gene expression in a zebrafish model in an oxidative environment induced by BPA exposure. Our findings will provide foundation for designing potential strategies to reduce BPA-induced neurotoxicity in the future.

## 2. Materials and Methods

### 2.1. Materials

WS was obtained from Yancheng in the Jiangsu Province of China. The WS was cut into 3–5 cm strips, ground, and sieved through a 40−80 mesh for subsequent experiments. BPA (purity, 99%) and DMSO-d6 were purchased from Sigma-Aldrich (St. Louis, MO, USA). Catalase (CAT) and superoxide dismutase (SOD) activity assay kits, malondialdehyde (MDA) content determination kit, and bicinchoninic acid (BCA) assay kit were purchased from Biyuntian Co. (Nanjing, China).

Preparing of LCCs from the WS. Before preparing LCCs, the WS was extracted in benzene:ethanol (2:1, *v*/*v*) using a Soxhlet extractor to remove pigments and wax. LCCs in the extracted WS (LCC-WS-A) were isolated and purified according to the standard protocol described by Bjorkman (1957) [20]. To obtain LCCs with a lower proportion of carbohydrates as the control group for LCC-WS-A to evaluate the oxidative ability, LCC-WS-A was subjected to a hydrothermal treatment in a stainless steel tube furnace. The stainless steel was heated to 180 °C for 10 min (including heating up time of 3 min) with a tube furnace, as described by Zhang et al. (2020) [21]. The treated LCC-WS-A (LCC-WS-B) was washed by deionized water to remove the degradation product and then freeze-dried to obtain the LCC-WS-B solid.

### 2.2. Analysis and Characterization of LCC Preparations

The chemical compositions of LCC preparations were analyzed according to the standard analytical procedure proposed by the National Renewable Energy Laboratory. The sugars in the acid hydrolysate during analysis were measured using HPAEC (Dionex ICS-5000, San Jose, CA, USA) with a PA10 column (2 mm × 250 mm) and a pulsed amperometric detector, as described by Zhou and Xu (2019) [22]. Specifically, the elution program consisted of an initial isocratic elution in 37 mM. NaOH from 0 to 20 min, followed 200 mM CH3COONa from 20 to 35 min, and finally equilibrated in 37 mM NaOH from 35 to 50 min.

The structural information of LCC preparations was obtained by quantitative 13C-NMR and 2D-HSQC NMR using NMR spectrometer (Bruker, Advance 600 MHz, Ettlingen German) equipped with a 5 mm BBO probe. The detailed procedures of sample preparation for NMR analysis are described in our previous study [15]. The amounts of various lignin substructures (β-*O*-4, β-β, and β-5) and LCC linkages (benzyl esters, benzyl ethers, and γ-esters) were calculated by the combination of quantitative 13C-NMR and 2D-HSQC NMR spectra, as described by Zhang and Gellerstedt (2007) [23].

### 2.3. Zebrafish Husbandry

In this study, the AB strain zebrafish (Danio rerio) obtained from the Institute of Hydrobiology of the Chinese Academy of Science (Wuhan, China) were used as the animal model. In the laboratory, the zebrafish were raised in a circulating culture system containing aerated tap water at 28 °C. During raising, dissolved oxygen in the water was controlled at >6 mg·L^−1^, and the photoperiod was 14 h of day and 10 h of night. Zebrafish were fed with newly hatched brine shrimp twice a day. Adult zebrafish were placed in cages, and fertilized eggs were collected. The fertilized eggs with normal development were selected under a microscope (Nikon, SMZ18, Tokyo, Japan) and washed with egg water (5 mM NaCl, 0.17 mM KCl, 0.33 mM CaCl2, and 0.33 mM MgSO4; pH 7.4) three times for the exposure experiment. Embryos were collected and cultured until 4 h post fertilization (hpf). The normal zebrafish embryos were selected for subsequent experiments. All the animal experiments were conducted in accordance with the guidelines for the care and use of laboratory animals of the Nanjing Institute of Environmental Sciences.

### 2.4. Effects of LCCs on the Hatching Rate of Zebrafish

The exposure concentration of BPA for zebrafish was 500 µg·L^−1^, which was selected according to a previous study by Molina et al. (2018) [24] and Kim SS et al. (2020) [25]. To investigate the protective effect of LCC on hatching rate, zebrafish eggs were exposed to control, BPA-treated (500 µg·L^−1^), BPA + LCC-WS-A (1 mg·L^−1^) co-treated and BPA + LCC-WS-B (1 mg·L^−1^) co-treated groups at 4 hpf until the 6 days of continuous exposure, and the number of zebrafish embryos in each of the four treatment groups was 30. Zebrafish husbandry for each group followed the aforementioned conditions. The embryo test solution was renewed every day to ensure the accuracy of solution concentration; activity of the subjects was continuously observed for timely removal of dead individuals. The hatching rate was calculated based on the number of hatched zebrafish from the total number of eggs laid.

### 2.5. Effects of LCCs on Zebrafish Behavior

To investigate the effects of LCCs on the behavior of BPA-treated zebrafish, 6 days-post-fertilization (dpf) larvae from the four treatment groups were randomly selected on a 24-well plate and placed in the DanioVision chamber (Noldus, Wageningen, the Netherlands). After acclimation for 10 min, free swimming activity in the continuous visible light (25 min) and in the dark (25 min) was monitored. EthoVision (Noldus, Wageningen, the Netherlands) was used to analyze the locomotor behavior and average speed during moving time.

### 2.6. Antioxidant Properties and Effects of LCCs on Oxidative Stress in Zebrafish

In vitro antioxidant properties of the LCC preparations were evaluated based on their scavenging activity against 2,2-diphenyl-1-picryl-hydrazyl (DPPH) and superoxide anion (O_2_^•−^) free radicals according to our previous study [15].

Explicitly, 2.0 mL of DPPH (0.2 mM) ethanol solutions (DPPH dissolving in anhydrous ethanol) and 2.0 mL of anhydrous ethanol were added to 2 mL of different concentrations of LCC solution, respectively. After 30 min, the absorbance of the mixtures was measured at 517 nm and the measured value was termed as A_sample_ and A_control_. In addition, 2.0 mL of anhydrous ethanol was added in 2 mL of DPPH (0.2 mM) ethanol solutions and absorbance of the mixtures was measured at 517 nm, termed as Ablank. The DPPH radical scavenging rate (P) was calculated by the following equation. The DPPH radical scavenging activity is expressed as the IC_50_, which are deduced directly from the plots of the concentration required for 50% inhibition of the free radical in the Y axis.

$$P(\%)=\frac{1-(A_{\mathrm{sample}}-A_{\mathrm{control}})}{A_{\mathrm{blank}}}\times 100\%$$

For O_2_^•−^scavenging assay, 1.0 mL of LCC solution (0.1−1 mg/mL) were mixed with 4.5 mL of Tris-HCl solution (0.05 mol/L with pH 8.2) in a tube and incubated at 25 °C for 20 min. Then, 0.5 mL of pyrogallol solution (45 mmol/L) was added to the tube. After the addition of pyrogallol solution, the absorbance of mixture was measured at 325 nm every 30 s, termed as O_sample_. Meanwhile, 0.5 mL of water was used to replace pyrogallol solution in the system. The supernate from this mixture was obtained to measure their absorbance at 325 nm, which is termed as O_control_. In addition, 1.0 mL of water ethanol was mixed with 4.5 mL of Tris-HCl solution (0.05 mol/L with pH 8.2) in a tube to measure the absorbance at 325 nm, termed as O_blank_. O_2_^•−^-radical scavenging rate (R) was calculated by the following equation. The O_2_^•−^-radical scavenging activity is expressed as the IC_50_, which are deduced directly from the plots of the concentration required for 50% inhibition of the free radical in the Y axis [26].

$$R(\%)=\frac{1-(O_{\mathrm{sample}}-O_{\mathrm{control}})}{O_{\mathrm{blank}}}\times 100\%$$

In vivo antioxidant properties of the LCC preparations were determined based on their scavenging activity against ROS in the BPA-treated zebrafish. Specifically, zebrafish were exposed to different treatments (control, BPA, BPA + LCC-WS-A co-treatment group and BPA + LCC-WS-B co-treatment group) for 6 days. Following staining with the diluted fluorescent probe 2-dichlorodihydrofluorescein diacetate at 28 °C for 20 min and washing with PBS three times, the fluorescence intensity was measured by confocal microscopy (Nikon, A1RHD25, Tokyo, Japan).

Six dpf larvae (*n* = 20) were collected from the four treatment groups, and their tissues were crushed by ultrasonication on ice (time 3 s, interval 6 s, amplitude 50%). The homogenate was centrifuged at 15,000 rpm for 10 min at 4 °C, and protein concentration in the supernatant was determined using the BCA assay kit (Beyotime Biotechnology, Nanjing, China). The supernatant of known protein concentration was placed on ice, and then SOD and CAT activity and MDA content were detected according to manufacturer’s instructions.

### 2.7. Neuroprotective Effects of LCCs in Zebrafish

A laser-scanning confocal microscope(Nikon, A1RHD25, Tokyo, Japan) was used to detect the expression intensity of green fluorescent protein (GFP) in 72 hpf transgenic (Tg) (Huc-GFP) larvae (*n* = 10) from the four treatment groups. The fluorescence intensity was then measured using ImageJ (National Institutes of Health, Bethesda, MD, USA).

### 2.8. Real-Time PCR

To determine the effects of LCCs on the transcription of key genes in BPA-treated zebrafish, mRNA levels of myelin basic protein (mbp) and synapsin (syn2a), involved in neuronal development, as well as of Cu/Zn-Sod and Cat, involved in oxidative stress response, were analyzed by real-time PCR. Specifically, total RNA from 20 live larvae in the four treatment groups was extracted and reversed-transcribed into cDNA, which was used as the template for PCR. The primer sequences of the tested genes (mbp, syn2a, Cu/Zn-Sod, and Cat) are listed in Supplementary Table S1.

### 2.9. Statistical Analysis

All experiments were independently repeated at least three times. Statistical differences between treatment groups were analyzed using one-way analysis of variance (ANOVA) followed by Student-Newman-Keuls post hoc test with SPSS 20.0 software (Chicago, IL, USA) or GraphPad Prism (GraphPad Software, San Diego, CA, USA) for multiple comparison tests. *p* < 0.05 was considered significant.

## 3. Results and Discussion

### 3.1. Composition and Molecular Weight of the LCC Preparations

In this study, LCC-WS-A in wheat stalk was extracted according to the method described by Bjorkman (1957) [27] which is the classic method to isolate the LCC from different biomass. The extracted LCC-WS-A was subjected to a short hydrothermal treatment at 180 °C for 10 min for partial degradation to obtain LCC-WS-B, which was used as the control preparation to evaluate the bioactivity of LCC-WS-A with different carbohydrate proportions. The obtained LCC-WS-A and LCC-WS-B contained elementals of C, H, O with amount of 61% and 56%, 32% and 33%, and 7% and 11%, respectively. As shown in Table 1, LCC-WS-A contained 42.5% lignin and 46.1% carbohydrates, with xylan as the predominant component. Following hydrothermal treatment, carbohydrates in LCC-WS-A were partially degraded; as such, in LCC-WS-B, the proportion of carbohydrates was reduced from 46.1% (LCC-WS-A) to only 25.9%, whereas the relative portion of lignin was increased from 42.5% (LCC-WS-A) to 64.2%. In addition, both LCC-WS-A and LCC-WS-B contained comparable amounts of polyphenols (3.3% and 3.1%, respectively). Regarding the molecular properties of the LCC preparations, the weight-average of molecular weight (Mw) and number-average of molecular weight (Mn) of LCC-WS-A (17,600 and 7890 g·mol^−1^, respectively) were higher than those of LCC-WS-B (9210 and 4520 g·mol^−1^, respectively) (Table 1).

BPA is a plastic additive, which is abundant in the environment, and it leads to neurological damage by disrupting the protein and lipid structures through free radical-mediated mechanisms both in vitro and in vivo [2]. Hence, many countries have now banned the production and sale of BPA-containing plastic products. In addition, various natural antioxidants derived from plants are being used as the protective substances to minimize the neurotoxic effects of BPA [8,9]. LCCs contain numerous bioactive substances such as lignin, carbohydrates, and polyphenols, which possess antioxidant activity against free radicals in vitro and ROS in vivo [14,15]. However, few previous studies have evaluated the neuroprotective effects of LCCs against BPA-induced oxidative stress, particularly in an in vivo zebrafish model. Therefore, in this study, LCCs from WS, a type of agricultural waste that is always burned, were isolated with the aims of (1) using WS as an alternative source of antioxidants against BPA neurotoxicity and (2) pursuing value-added application of WS waste. In plant cell walls, various amounts of different carbohydrates are linked with lignin by covalent bonds (PhGlc, BE, and Est) to form LCCs [28,29]. To verify the association of LCC structure with its antioxidant activity, LCCs extracted from WS (LCC-WS-A) were subjected to a short hydrothermal treatment to partially degrade the carbohydrates, and the resulting LCC (LCC-WS-B) was used as the control.

### 3.2. Structural Properties of the LCC Preparations

**Figure 1:**
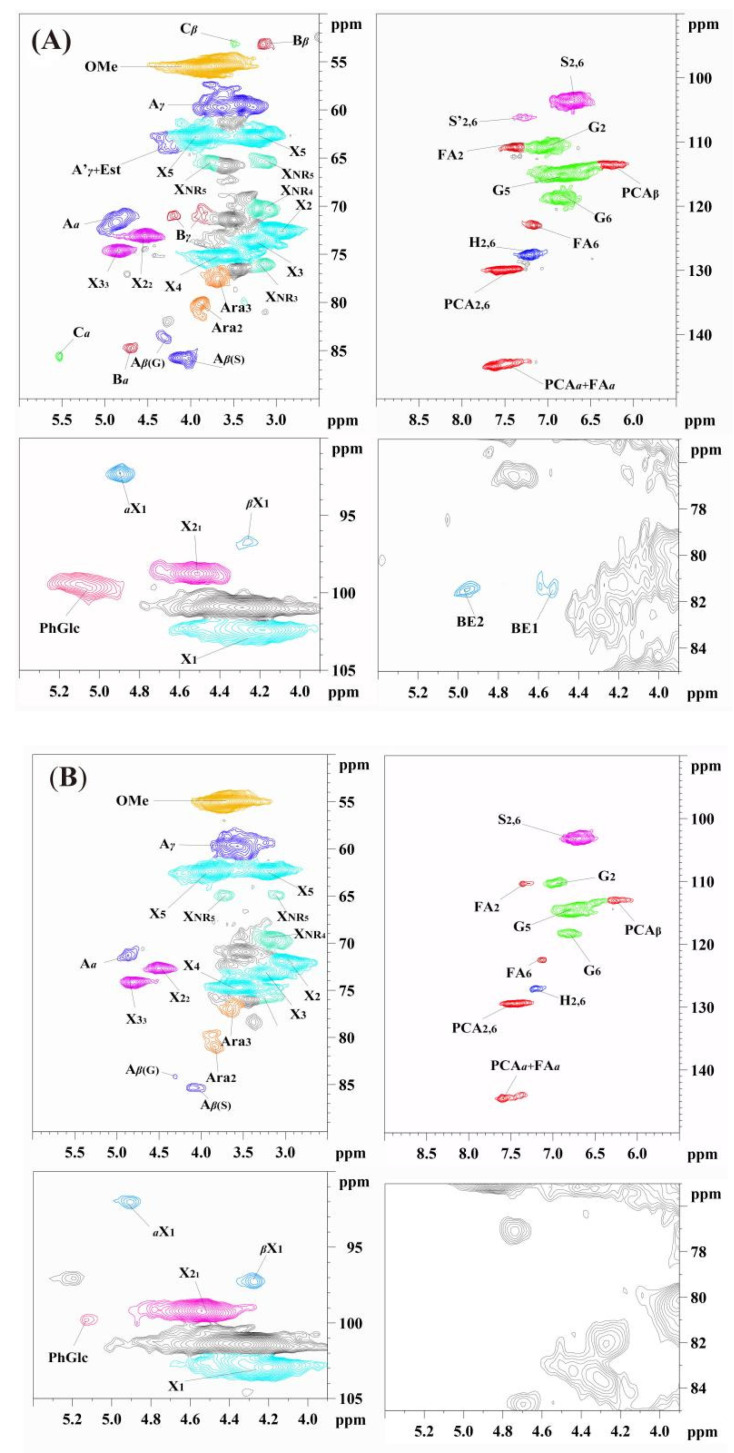
2D-HSQC NMR spectra of LCC-WS-A (**A**) and LCC-WS-B (**B**).

To analyze the detailed structure of LCC-WS-A and LCC-WS-B, 13C-NMR and 2D-HSQC NMR were performed for identifying the linkages and substructures in LCCs and their quantitative estimates. In the side-chain regions (δC/δH 90–50/6.0–2.5 ppm) of the 2D-HSQC spectra (Figure 1) of LCC-WS-A and LCC-WS-B, the signals corresponding to the β-O-4 linkages (A substructure) were identified by its C_γ-_H_γ_, C_α_-H_α_, and C_β_-H_β_ signals at δC/δH 59.8/3.75, 71.9/4.91, and 83.6–85.8/4.39–4.13, respectively. The substructures of β-β (resinols, B) and β-5 (phenylcoumarans, C) linkages were also observed in the 2D-HSQC spectra of LCC-WS-A, which were identified based on their corresponding Ca-Ha signals at δC/δH 84.9/4.69 and 86.8/5.49, respectively. By contrast, the signals of β-β and β-5 linkages were absent in the LCC-WS-B spectra. In the aromatic regions (δC/δH 160–90/8.0–6.0) of the 2D-HSQC spectra of LCC-WS-A and LCC-WS-B, the S, G, and H subunits were detected based on their signals. Specifically, the signals of C_2,6_-H_2,6_ corresponding to the S and H units were observed at δC/δH 104.1/6.72 and 127.8/7.22, respectively. In addition, the signals of C_2_-H_2_, C_5_-H_5_, and C_6_-H_6_ corresponding to the G unit were observed at δC/δH 110.9/7.02, 114.5/6.71, and 119.0/6.81, respectively. The representative units of gramineous lignin, namely p-coumarate (PCA) and ferulate (FA), were also detected in the LCC-WS-A and LCC-WS-B spectra. The prominent signals corresponding to PCA substructures are observed at δC/δH 130.5/7.41, 144.6/7.51, and 113.9/6.31, which are attributed to C_2,6_-H_2,6_, C_3,5_-H_3,5_, C_α_-H_α_, and C_β_-H_β_ of PCA, respectively. The signals for the C_6_-H_6_ of FA are observed at δC/δH 123.9/7.09 in both LCCs spectra.

To analyze changes in the substructures and linkages of LCCs, 13C-NMR and 2D HSQC NMR were combined and used to quantify their amounts (per 100 Ar (C900)) (Supplementary Figure S1). As shown in Table 2, LCC-WS-A contained the highest amount of each substructure (21.9/100 Ar of β-*O*-4, 5.5/100 Ar of β-β, and 2.3/100 Ar of β-5). Meanwhile, LCC-WS-B contained only 14.1/100 Ar of β-*O*-4. Following the hydrothermal treatment of LCC-WS-A, the S:G ratio increased from 0.8 to 1.9.

Furthermore, the accredited linkages of phenyl glycoside (PhGlc), benzyl ether (BE), and γ-ester (Est) were identified in the 2D-HSQC spectra of LCC-WS-A (Figure 1). Specifically, PhGlc showed a signal at δC/δH 99.1/5.09. Two kinds of BE linkages were noted; a C1-linkage between the α-position of lignin and the primary OH group of carbohydrates (BE1) and a C_2_-linkage between the α-position of lignin and the secondary OH group of carbohydrates (BE2) showed signals at δC/δH 81.2/4.51 and 81.2/4.93, respectively. In addition, the ester bond in LCC-WS-A was identified based on its C_γ_–H_γ_ signal at δC/δH 65−62/4.5−4.0 (Est), which likely overlapped with the structure of γ-acylated β-O-4 (A’γ). In LCC-WS-A spectra, PhGlc and Est were detected based on the signals of C-H, but the signals for both BE1 and BE2 were absent. According to the quantitative results (Table 2), LCC-WS-A contained 22.7/100 Ar of LCC linkages, with 2.9/100 Ar of BE, 13.5/100 Ar of PhGlc, and 6.3/100 Ar of Est, whereas LCC-WS-B contained only 10.2/100 Ar of LCC linkages, with 4.3/100 Ar of PhGlc and 5.9/100 Ar of Est.

In the present study, LCC-WS-A was the carbohydrate-rich LCC characterized by its higher carbohydrate content than lignin content. Following hydrothermal treatment, galactan and mannan were degraded owing to their branched structure in the xylan skeleton of LCC-WS-A, resulting in a lignin-rich LCC (LCC-WS-B) [21]. Although some carbohydrates were degraded during the hydrothermal treatment, xylan was the major sugar in both LCC-WS-A (87% of total carbohydrates) and LCC-WS-B (90% of total carbohydrates). Moreover, owing to carbohydrate degradation, the Mw of LCC-WS-A also decreased. In addition to carbohydrates and lignin, LCC-WS-A also contained polyphenols in its structure. Polyphenols are regarded as important biopolymers with antioxidant activity [30]. Hence, polyphenols in LCC-WS-A may imbue it with a stronger antioxidant activity than lignin or carbohydrates alone. Fortunately, the polyphenols detected in LCC-WS-A were not obviously degraded during hydrothermal treatment. Regarding the structure, LCC-WS-A contained both the basic substructures of β-*O*-4, β-β, and β-5 as well as the typical LCC linkages of PhGlc, BE, and Est. When carbohydrates in LCC-WS-A were degraded following hydrothermal treatment, some LCC substructures and linkages were also partially degraded. Therefore, LCCs can be degraded by hydrothermal treatment, suggesting that this can be regarded as the candidate method to synthesize structurally diverse LCCs. Finally, the antioxidant activity of LCC-WS-A and LCC-WS-B is indeed associated with their structural characteristics.

### 3.3. Effects of LCCs on the Survival Rate and Hatching Rate of Zebrafish

**Figure 2:**
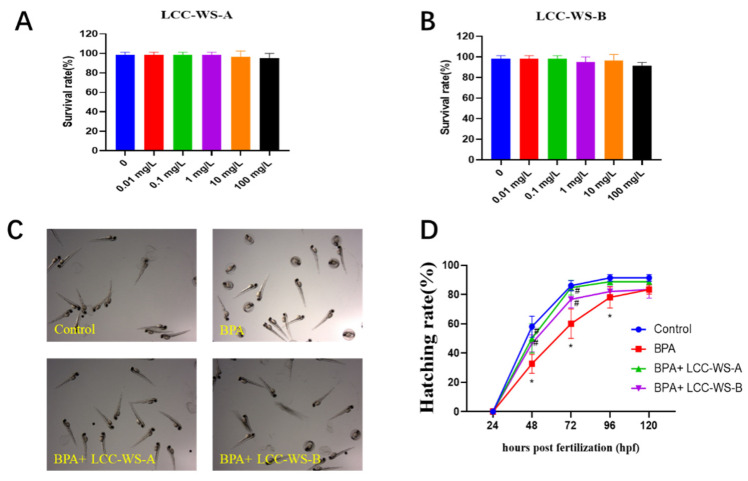
Effects of LCC-WS-A and LCC-WS-B on BPA toxicity in developing zebrafish. Survival rate following LCC-WS-A (**A**) and LCC-WS-B (**B**) treatment. Representative images of the hatched zebrafish larvae in different treatment groups at 72 hpf (**C**). Statistical results of the hatching rate of zebrafish (**D**). * *p* < 0.05, compared with the control group. # *p* < 0.05, compared with BPA-treated group.

The survival rate of zebrafish treated with the LCC preparations was analyzed to evaluate the toxicity of LCCs. The survival rate of zebrafish treated with low concentrations (0.01−10 mg·L^−1^) of LCC-WS-A and LCC-WS-B remained near 100% (Figure 2A,B). However, the survival rate decreased slightly (−5%) when the concentration of LCC-WS-A or LCC-WS-B increased from 10 to 100 mg·L^−1^. The above results indicate that concentrations below 100 mg·L^−1^ were not toxic to zebrafish.

Although the hatching rate of zebrafish embryos decreased following BPA treatment, this decrease was recovered when the BPA-treated zebrafish embryos were further treated with the LCC preparations (Figure 2C). Specifically, at 72 hpf, the hatching rate of zebrafish embryos decreased from 82.5% to 60% following treatment with BPA (500 µg·L^−1^), but this rate increased to 84.67% and 76.67% following further treatment with LCC-WS-A and LCC-WS-B (1 mg·L^−1^), respectively (Figure 2C,D).

As the morphological and molecular characteristics of tissues and organs in zebrafish are similar to those in other vertebrates, including humans, they are regarded as a suitable animal model for evaluating the toxicity and safety of chemical compounds [19,21]. As shown in this study, BPA was toxic to zebrafish embryos at the hatching stage, and this toxicity was alleviated by the cotreatment of LCCs and BPA, albeit without significant differences. This result indicates that LCCs from WS can indeed reduce BPA toxicity in zebrafish.

### 3.4. Effects of LCC-WS-A and LCC-WS-B on the Behavior of BPA-Treated Zebrafish

**Figure 3:**
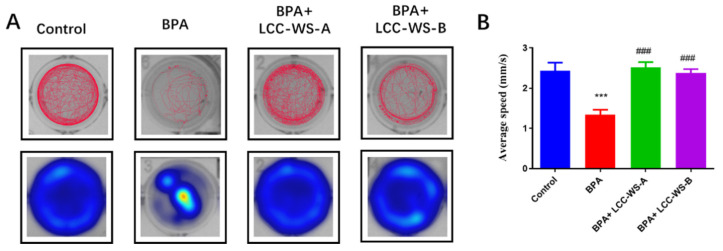
Effects of different treatments on the locomotor behavior of 6 dpf zebrafish larvae. Representative movement tracks and heat maps (**A**). Average speed (**B**). *** *p* < 0.001, compared with the control group. ### *p* < 0.001, compared with the BPA-treated group.

To investigate effects on the locomotor behavior, zebrafish larvae were treated with BPA, BPA + LCC-WS-A, and BPA + LCC-WS-B for 6 days. The locomotor behavior of zebrafish larvae in the BPA-treated group (1.34 mm·s^−1^) was significantly inhibited compared with that of zebrafish larvae in the control group (2.44 mm·s^−1^) (Figure 3). However, the co-treatment of BPA + LCC-WS-A and BPA + LCC-WS-B significantly restored the abnormal locomotor behavior of zebrafish larvae caused by BPA to the control level. Specifically, the average speed during moving time was 2.52 mm·s^−1^ in the LCC-WS-A-treated group and 2.37 mm·s^−1^ in the LCC-WS-B-treated group, indicating that LCC-WS-A was more effective than LCC-WS-B in restoring the BPA-altered locomotor behavior of zebrafish.

To understand whether LCCs can reduce BPA neurotoxicity, the locomotor behavior controlled by the nervous system of zebrafish was tested after 6 days of BPA treatment and BPA + LCC cotreatment. The locomotor behavior of zebrafish larvae (average speed) was inhibited following BPA treatment compared with that following control treatment (about 55.0% of the control group). Meanwhile, LCC-WS-A totally restored the BPA-inhibited locomotor behavior of zebrafish, as evidenced by normal total traveled distance and average speed, and LCC-WS-A (about 103.2% of the control group) was better than LCC-WS-B (about 97.3% of the control group) in restoring this behavior. Hence, the LCC with a higher carbohydrate content showed a better ability to reduce potential BPA neurotoxicity than the LCC with a lower carbohydrate content.

### 3.5. In Vitro and In Vivo Antioxidant Properties of LCCs and their Protective Effects against BPA-Induced Oxidative Damage in Zebrafish

**Figure 4:**
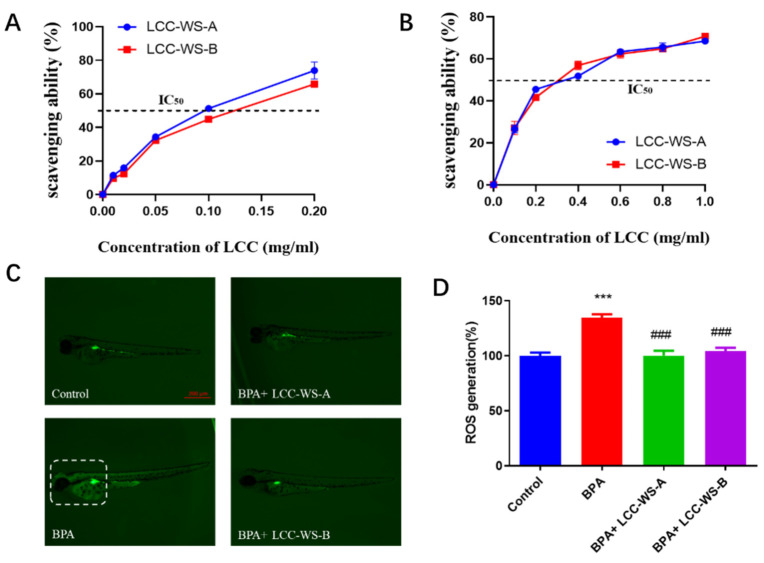
Free radical scavenging activity of LCC-WS-A and LCC-WS-B against DPPH (**A**) and O_2_^•−^ radicals (**B**). Fluorescent images of reactive oxygen species in zebrafish larvae in different treatments groups (**C**). Statistical analysis of fluorescence (**D**). *** *p* < 0.001, compared with the control group. ### *p* < 0.001, compared with the BPA-treated group.

The in vitro antioxidant properties of LCC-WS-A and LCC-WS-B were evaluated based on their scavenging activity against DPPH and O_2_^•−^ radicals. LCC-WS-A showed stronger ability to scavenge DPPH than LCC-WS-B (Figure 4A). Specifically, the IC50 (the concentration of LCCs preparation for scavenging 50% of the initial radicals in vitro) of LCC-WS-A (0.09 mg·mL^−1^) was lower than that of LCC-WS-B (0.13 mg·mL^−1^). LCC-WS-A and LCC-WS-B showed comparable scavenging activity against O_2_^•−^ radicals, with the IC50 values of 0.3 mg·mL^−1^ (Figure 4B).

To evaluate the in vivo antioxidant properties of the LCC preparations, their ability to scavenge ROS was evaluated in a zebrafish model. Compared with the control group (normal zebrafish), the BPA-treated group showed significant ROS accumulation, particularly in the head. Considering 100% as the normal value, ROS level in the BPA-treated group reached 134.67% (Figure 4C). Meanwhile, the LCC-WS-A-treated and LCC-WS-B-treated groups showed significantly decreased ROS levels from 134.67% (BPA-treated group) to 100.3% and 104.3%, respectively.

**Figure 5:**
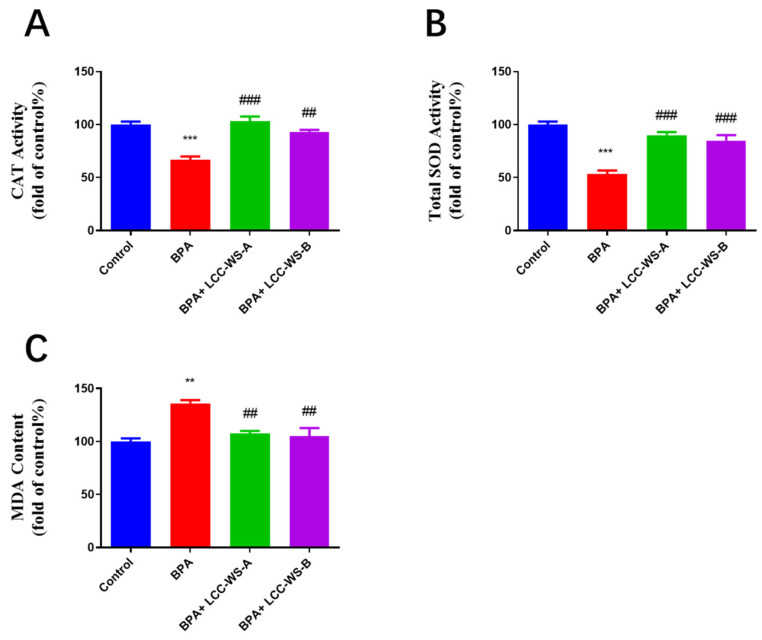
Effects of LCC-WS-A and LCC-WS-B on BPA-induced oxidative stress in zebrafish. LCC-WS-A and LCC-WS-B increased the activity of CAT (**A**) and SOD (**B**) but decreased the content of MDA (**C**). ** *p* < 0.01, *** *p* < 0.001, compared with the control group. ## *p* < 0.01, compared with BPA-treated alone. ### *p* < 0.001, compared with BPA-treated alone.

To elucidate the protective effects of LCCs against BPA-induced oxidative stress, the activity of CAT and SOD and content of MDA in zebrafish treated with BPA, BPA + LCC-WS-A, and BPA + LCC-WS-B were measured. Compared with the control group, the BPA-treated group showed significantly suppressed activity of CAT and SOD and significantly increased content of MDA, suggesting that BPA led to oxidative damage in zebrafish (Figure 5A–C). Meanwhile, this BPA-induced oxidative damage was alleviated in the LCC-WS-A-treated and LCC-WS-B-treated groups, as evidenced by the increased activity of CAT and SOD and decreased content of MDA.

Many studies have shown that BPA neurotoxicity is related to oxidative stress. For instance, Pang et al. (2019) [5] reported that BPA and its substitutes increased ROS levels in HT22 (nerve cells) cells, resulting in apoptosis and neurotoxicity; this is consistent with our results. Through in vivo experiments, we found that BPA produced neurotoxic effects by inducing oxidative stress in zebrafish, thereby significantly increasing ROS levels. In this study, LCC-WS-A and LCC-WS-B showed satisfactory free radical scavenging activity in vitro and antioxidant activity against ROS in vivo. Importantly, both LCC preparations exhibited potent neuroprotective effects under BPA-induced oxidative stress in zebrafish. The antioxidant activity of LCC-WS-A and LCC-WS-B may be attributed to their lignin, carbohydrate, and polyphenol components, all of which exhibit free radical scavenging activity [14,15]. Notably, however, the antioxidant activity of LCC-WS-A, which contained more carbohydrates, was stronger than that of LCC-WS-B, which contained more lignin. This result is consistent with the report of Zhao et al. (2015) [31] that the hemicellulose fraction of LCCs with different monosaccharide compositions demonstrated superior antioxidant capacity than the lignin-rich fraction. According to Zhao et al. (2015) [9], the antioxidant ALA showed potent protective effects against BPA-induced neurotoxicity in astrocytes of C8-D1A mice as well as against neurochemical and neurobehavioral defects in C57BL/6J male mice through the mechanisms of free radical scavenging and antioxidant enzyme regulation. Hence, details of LCC-induced changes in the activities of enzymes that maintain the redox status and prevent oxidative damage following the BPA exposure of zebrafish should be analyzed.

In this study, the activity of SOD and CAT in BPA-treated zebrafish larvae was significantly decreased compared to that in control zebrafish larvae, indicating that BPA reduced the antioxidant potential by inhibiting the activity of redox enzymes. Following LCC treatment, however, the activity of SOD and CAT was recovered to the normal level, perhaps owing to the antioxidant activity of LCC against ROS [32]. These results are consistent with the report by Zhao et al. (2015) [15] that LCCs from bamboo and poplar increased the activity of SOD and CAT in zebrafish following H_2_O_2_-induced oxidative damage. Notably, however, the degree of recovery with LCC-WA-B treatment was slightly lower than that with LCC-WA-A treatment, perhaps owing to the weaker antioxidant activity of the former than that of the latter. As SOD and CAT activity is the key factor representing the degree of antioxidant capacity or oxidative damage in zebrafish [33,34,35], LCCs with a high carbohydrate content may show a better ability to minimize oxidative damage.

### 3.6. Effects of LCC-WS-A and LCC-WS-B on Gene Expression in Tg Zebrafish

**Figure 6:**
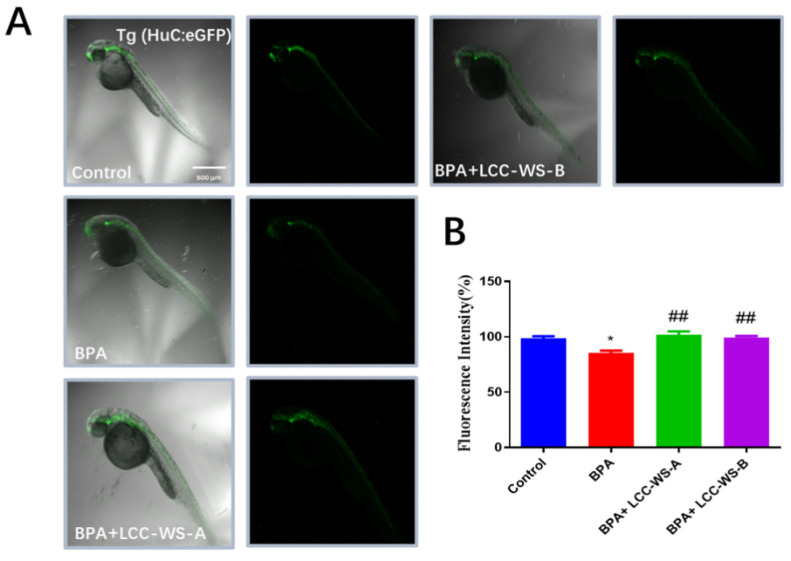
Effects of LCC-WS-A and LCC-WS-B on neurogenesis in Tg (HuC-GFP) zebrafish. Green fluorescence images of neurogenesis in 72 hpf Tg (HuC-GFP) zebrafish (**A**) and statistical analysis of fluorescence (**B**) (*n* = 10 in each group). * *p* < 0.05, compared with the control group. ## *p* < 0.01, compared with the BPA-treated group.

To directly study the effects of LCC-WS-A and LCC-WS-B on neuronal development, we selected a Tg zebrafish line (Huc-GFP). Following the exposure of Huc-GFP zebrafish to different treatments (control, BPA, BPA + LCC-WS-A, and BPA + LCC-WS-B), the expression of GFP in the brain and spinal cord was significantly decreased in the BPA-treated group but significantly restored to normal in the LCC-WS-A–treated and LCC-WS-B-treated groups (Figure 6).

**Figure 7:**
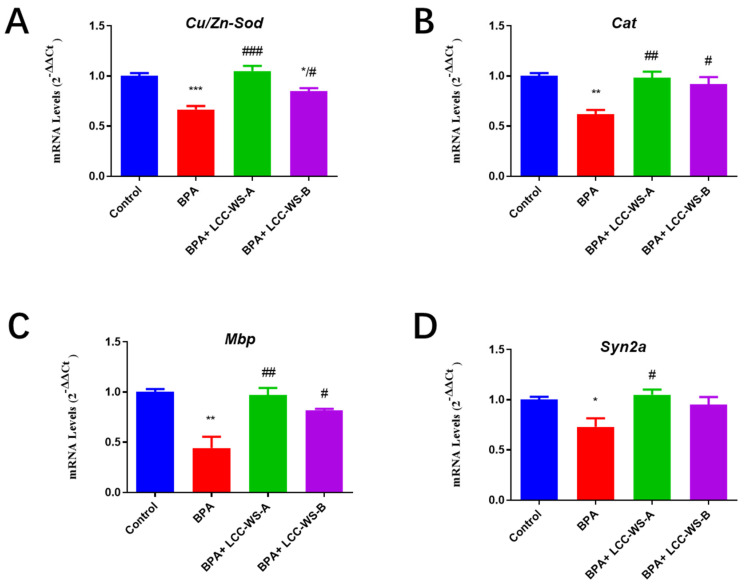
Effects of LCC-WS-A and LCC-WS-B on the expression of oxidative stress- and neurogenesis-related genes in BPA-treated zebrafish. Cu/Zn-Sod (**A**), cat (**B**), mbp (**C**) and syn2a (**D**). * *p* < 0.05, ** *p* < 0.01, *** *p* < 0.001, compared with control groups. # *p* < 0.05, ## *p* < 0.01, ### *p* < 0.001, compared with the BPA-treated group.

To gain insights into the antioxidant activity of LCCs in the nervous system, the expression levels of genes related to oxidative stress (Cu/Zn-Sod and cat) and neurogenesis (mbp and syn2a) were analyzed by real-time PCR. The expression of Cu/Zn-Sod and Cat in zebrafish was significantly inhibited by BPA treatment but significantly restored to the normal level by LCC-WS-A treatment (Figure 7A,B). LCC-WS-B significantly restored the expression of Cat to the normal level; however, although it restored the expression of Cu/Zn-Sod, the level did not return to normal (Figure 7A,B). To further explore the molecular mechanisms underlying BPA-induced nerve damage in zebrafish, we detected the expression of two key genes related to neurogenesis. Mbp expression in oligodendrocytes is commonly used as a biomarker for axonal myelination during neurogenesis in zebrafish [36] and humans [37]. Syn2a is a biomarker for synaptic formation in mammals and plays pivotal roles in synaptogenesis and neurotransmitter release [38]. Moreover, the expression of genes (mbp and syn2a) related to neurogenesis in larvae was examined. Compared with that in the control group, the expression of mbp and syn2a was significantly inhibited in the BPA-treated group, suggesting neurotoxicity (Figure 7). LCC-WS-A significantly restored the expression of mbp and syn2a. Meanwhile, although LCC-WS-B restored the expression of mbp and syn2a, the effect was not significant. These results suggest that LCCs can alleviate the neuronal damage caused by BPA.

BPA-induced inhibition of mbp expression may lead to the loss of myelin and disruption of neuronal function. Syn2a downregulation may affect synaptogenesis and neuronal differentiation. In addition, BPA significantly inhibited the expression of Cu/Zn-Sod and cat mRNA. Interestingly, however, cotreatment with LCC-WA-A or LCC-WA-B significantly augmented the expression of all tested genes (mbp, syn2a, Cu/Zn-Sod, and cat) and protected the nerve to some extent, which is a crucial finding. Gene expression levels are positively linked to neurogenesis and larval growth [17]. In this study, LCCs with a high carbohydrate content showed a better ability to restore the transcription of nervous system genes inhibited by BPA. Consistent with changes in the expression of neurodevelopment-related genes, BPA exposure significantly reduced the GFP fluorescence in the brain and spinal cord of Huc-GFP zebrafish; however, LCCs recovered this effect. Together, these results suggest that LCCs exhibit potent protective effects against BPA-induced neurotoxicity.

## 4. Conclusions

In conclusion, carbohydrate-rich and lignin-rich LCCs were successfully prepared and their composition and structure were analyzed. BPA exposure negatively affected locomotor behavior, CAT and SOD activity, larval development, and gene expression in zebrafish larvae. The LCC preparation almost completely recovered from these negative effects by relieving oxidative stress compared to the control group. The carbohydrate-rich LCC showed a stronger antioxidant activity than the lignin-rich LCC in BPA-treated zebrafish, indicating its potential as a neuroprotective agent.

## Figures and Tables

**Table 1:** Composition and molecular weight of LCC-WS-A and LCC-WS-B.

Preparation	Lignin (%)	Cellulose (%)	Hemicellulose (%)				Molecular Weight			Polyphenols (%)
			Xylan	Arabinan	Galactan	Mannan	M_w_ (g·mol^−1^)	M_n_ (g·mol^−1^)	PDI	
LCC-WS-A	42.5	3.4	40.5	1.9	0.2	0.1	17,600	7890	2.2	3.3
LCC-WS-B	64.2	1.6	23.5	0.8	0	0	9210	4520	2.0	3.1

M_w_: weight-average of molecular weight; M_n_: number-average of molecular weight; PDI: polydispersity index (M_w_/M_n_).

**Table 2:** Amounts (semi-quantitative data) of lignin substructures and lignin-carbohydrate linkages in LCC-WS-A and LCC-WS-B (per 100 Ar).

Characteristics	LCC-WS-A	LCC-WS-B
Lignin interunit linkages		
β-O-4 aryl ethers (A)	21.9	14.1
Resinols (B)	5.5	-
Phenylcoumarans, β-5 (C)	2.3	-
S:G ratio ^a^	0.8	1.9
LCC linkages		
Benzyl ether (BE)	2.9	-
Phenyl glycoside (PhGlc)	13.5	4.3
Est ^b^	6.3	5.9
Total ^c^	22.7	10.2

^a^: Calculated from the 2D HQSC spectra as following formula: S/G = I_103−108_/_6.4−7.0_ ppm/2/I_108−114_/_6.9−7.3_ ppm; ^b^: γ-ester; ^c^: BE+PhGlc+Est.

## Data Availability

Data is contained within the article or supplementary files.

## Supplementary Materials

The following are available online at https://www.mdpi.com/article/10.3390/antiox10101640/s1, Figure S1: The ^13^C NMR spectra of LCC-WS-A (red) and LCC-WS-B (black), Table S1: Sequences of primers for the genes tested.
