# Supplementary material for: Protective Effects of Lignin-Carbohydrate Complexes from Wheat Stalk against Bisphenol a Neurotoxicity in Zebrafish via Oxidative Stress
Source: Antioxidants (Basel). 2021 Oct 18;10(10):1640. doi: 10.3390/antiox10101640 (PMC8533324; doi:10.3390/antiox10101640)
Supplement: Supplementary file 1 [file antioxidants-10-01640-s001.zip › antioxidants-1348427-supplementary.pdf]

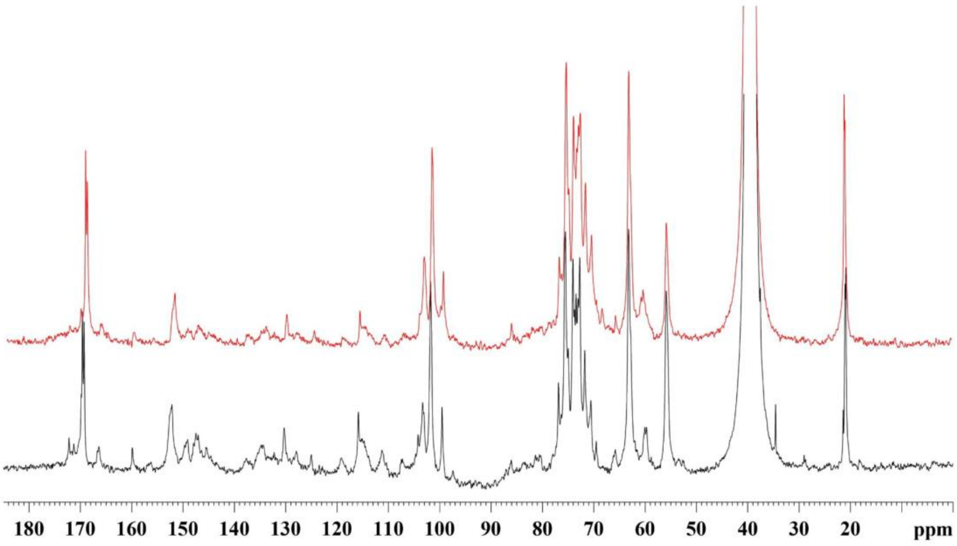

Figure S1. The ^{13}C NMR spectra of LCC-WS-A (red) and LCC-WS-B (black).

Table S1. Sequences of primers for the genes tested.

Name	Gene no.	Primer sequence
Cu/Zn-Sod	NM_131294.1	Forward: 5′-GTCGTCTGGCTTGTGGAGTG-3′<br>Reverse: 5′-TGTCAGCGGGCTAGTGCTT-3′
Cat	NM_130912.2	Forward: 5′-AGGGCAACTGGGATCTTACA-3′<br>Reverse: 5′-TTTATGGGACCAGACCTTGG-3′
mbp	AY860977	Forward: 5′-AATCAGCAGGTTCTTCGGAGGAGA-3′<br>Reverse: 5′-AAGAAATGCACGACAGGGTTGACG-3′
syn2a	NM_001002597	Forward: 5′-GTGACCATGCCAGCATTTC-3′<br>Reverse: 5′-TGGTTCTCCACTTTCACCTT-3′
β-actin	AF025305	Forward: 5′-ACAGGGAAAAGATGACACAGATCA-3′<br>Reverse: 5′-CAGCCTGGATGGCAACGTA-3′
